# Effect of Mechanical Surface Treatment on Shear Bond Strength of Orthodontic Brackets to 3D Printed and Milled CAD/CAM Provisional Materials: An In Vitro Study

**DOI:** 10.3390/jfb15120358

**Published:** 2024-11-25

**Authors:** Abdulaziz A. Alzaid, Khalid K. Alanazi, Lulu A. Alyahya, Maha N. Alharbi, Hatem Alqarni, Mohammed Alsaloum, Hayam A. Alfallaj, Ghada S. Alotaibi

**Affiliations:** 1 Restorative and Prosthetic Dental Sciences Department, College of Dentistry, King Saud bin Abdulaziz University for Health Sciences, Riyadh 11481, Saudi Arabia; qarnih@ksau-hs.edu.sa (H.A.); saloumm@ksau-hs.edu.sa (M.A.); fallajh@ksau-hs.edu.sa (H.A.A.); 2 King Abdullah International Medical Research Center, Riyadh 11481, Saudi Arabia; alyahyalulu@gmail.com (L.A.A.); maaha.naser@gmail.com (M.N.A.); ghada.alotibi@gmail.com (G.S.A.); 3 Conservative Dental Science Department, College of Dentistry, Prince Sattam bin Abdulaziz University, Al-kharj 11942, Saudi Arabia; kk.alanazi@psau.edu.sa; 4 College of Dentistry, King Saud bin Abdulaziz University for Health Sciences, Riyadh 11481, Saudi Arabia; 5 King Abdulaziz Medical City, Ministry of the National Guard—Health Affairs, Riyadh 11426, Saudi Arabia

**Keywords:** provisional, crown, CAD/CAM, bracket, bonding

## Abstract

The aim of the study is to assess the impact of mechanical surface treatments on the shear bond strength (SBS) of orthodontic brackets bonded to three-dimensional (3D) printed and milled CAD/CAM provisional materials. Sixty cylindrical samples were fabricated for each provisional material. Samples were treated with one of the following surface treatments: aluminum oxide airborne particle abrasion, diamond bur rotary instrument roughening, and phosphoric acid etching (control). Stainless steel brackets were bonded to the samples, and then SBS was tested using a universal testing machine. SEM and digital microscopy were utilized to examine the bonding interface and the failure modes. Two-way ANOVA, one-way ANOVA, Tukey’s HSD, and independent sample *t*-tests were used for statistical analysis. Results revealed significant differences in SBS between 3D printed and milled samples and significant differences in SBS among most surface treatments, with rotary instrument roughening resulting in the highest values for 3D printed, while airborne particle abrasion leading for milled samples. Digital microscopy indicated that more adhesive remained on 3D-printed samples. SEM analysis revealed varying surface roughness across treatments. Based on the findings of this study, it can be concluded that different surface treatments improve the bonding of orthodontic brackets to provisional crowns.

## 1. Introduction

As the demand for adult orthodontics continues to grow [1], practitioners are increasingly faced with patients who have structurally compromised teeth or highly restored dentitions, which often involve existing crowns, fixed partial dentures, and implants. Effectively managing these cases requires a careful interdisciplinary treatment plan that incorporates the expertise of multiple dental specialties. A critical challenge in such scenarios is the bonding of orthodontic brackets to existing restorations, which is crucial for the success of the treatment, as well as the subsequent de-bonding procedures, both of which can potentially compromise the integrity of permanent prostheses [1,2,3]. To mitigate these risks, it is widely recommended to utilize provisional crowns as substitutes for permanent restorations. Provisional crowns (PC) serve as long-term temporary prostheses, providing essential protection to the teeth and surrounding soft tissues. Additionally, they restore both function and esthetics, ensuring the preservation of the dentition until the completion of orthodontic treatment. This approach allows for the safe management of restorative materials during treatment, contributing to the overall success of dental care [2,3].

Provisional crowns can be fabricated through conventional methods using materials such as bis-acryl, composite resin, and acrylic resin based on methacrylate (MMA), polymethyl methacrylate (PMMA), polyethyl methacrylate (PEMA), or urethane dimethacrylate (UDMA) or by utilizing prefabricated temporary options, such as polycarbonate crowns, to temporarily restore prepared teeth [3]. Alternatively, computer-aided design and computer-aided manufacturing (CAD/CAM) technology can be employed to fabricate provisional crowns with enhanced precision and superior marginal integrity [4]. Utilizing CAD/CAM technologies to fabricate provisional crowns can be categorized into two methods: subtractive manufacturing (SM), such as milling, and additive manufacturing (AM), such as three-dimensional (3D) printing [5]. SM, particularly in the form of CAD/CAM milling, involves removing material from a prefabricated block or disc, [6,7,8] resulting in a structure with higher filler content that was originally packed under higher temperatures and pressure, with lower porosity, voids, and residual monomers compared to 3D printing and conventional resins [9]. However, this method has several limitations: it leads to the wastage of raw material, the milling tools have a limited lifespan due to wear and abrasion, and the precision of the milled product depends on factors such as the movement, type, and size of the milling tool. These factors often lead to inferior fit and marginal adaptation, especially for complex designs [5,10].

In contrast, AM, commonly known as 3D printing, has gained attention in the literature for its ability to overcome many of the disadvantages associated with milling [11]. Three-dimensional printing is a manufacturing process that builds objects layer by layer, allowing for greater precision and customization [12]. Several AM techniques are currently used in dentistry, with the most common being stereolithography (SLA), selective laser sintering (SLS), fused deposition modeling (FDM), digital light processing (DLP), PolyJet, and bioprinting [13]. With continuous improvements in the quality of 3D printing, its application in producing dental restorations has expanded, particularly in complex cases and for patients with special needs [14,15,16,17]. AM offers significant advantages over SM, including the ability to create restorations with fine internal geometries and complex prostheses while minimizing material waste [18,19]. It also exhibited higher fracture resistance [20]. In a recent systematic review in 2022, mechanical properties, excluding microhardness, toughness, and resilience, of 3D printed provisional materials were found to be superior [21]. Thus, the CAD/CAM printed or milled materials have superior strength, durability, wear resistance, superior fit, aesthetics, and longevity, making them suitable for PC manufacture.

Bonded orthodontic brackets must have sufficient bond strength to withstand orthodontic forces. A weak bond between the brackets and provisional materials can lead to a high failure rate, adversely affecting the cost, efficiency of orthodontic therapy, and patient comfort [22]. Many factors influence the bond strength between brackets and provisional materials, including the type of provisional material, the adhesive material, the duration after bonding, and the oral environment temperature [23,24,25,26,27,28,29]. Now, there are also other treatment options without the use of brackets, such as aligners.

Various treatments have been employed to enhance the bond strength of orthodontic brackets to provisional crowns. Chemical methods include the application of phosphoric acid or hydrofluoric acid at varying concentrations and durations [30,31,32]. Mechanical techniques involve sandblasting and surface roughening with diamond or carbide burs of different coarseness [22,31,32,33,34,35]. Additionally, laser treatments, such as CO2 laser, have been explored to further improve bonding strength [35,36]. Studies have suggested that brackets exhibited different shear bond strength (SBS) under various conditions [34,37]. It has been recommended that a minimum bond strength of 6–8 MPa between orthodontic brackets and teeth to ensure effective clinical orthodontic movement [37,38].

The quality of the bond between the orthodontic bracket and its substrate can be assessed by evaluating the residual adhesive remaining on the substrate surface after de-bonding through the use of the Adhesive Remnant Index (ARI) [30,32]. This index indicates the nature of the bond failure, distinguishing between adhesive failure (at the adhesive and provisional crown interface) and cohesive failure (at the adhesive and bracket interface). The distinct surface morphologies of the bracket adhesive interface can be analyzed using scanning electron microscopy (SEM) [22,39,40].

The aim of this study is to evaluate the SBS of orthodontic brackets to 3D printed and milled provisional crown materials following mechanical surface treatments. The null hypothesis is that there will be no difference in SBS between 3D printed and milled crown materials, regardless of the mechanical surface treatment.

## 2. Materials and Methods

Samples were designed as cylinders measuring (15 mm in diameter and 15 mm in height) using 3D design software (Meshmixer 3.5.0; Autodesk, San Francisco, CA, USA). A Standard Triangle Language (STL) file was created to fabricate all samples tested in this study. Sixty cylinders were 3D printed using a DLP 3D printer (Asiga Max UV; Asiga, Alexandria, Australia) with micro-filled hybrid provisional resin material (FREEPRINT TEMP; Detax, Ettlingen, Germany) (Table 1, Figure S1). The printing parameters included 45-degree orientation, 50 μm layer thickness, and 385 nm wavelength. Upon completion of printing, samples were removed carefully from the printing platform using a scraper and pre-cleaned in an ultrasonic bath of 99.9% isopropyl alcohol for 4 min, which was followed by a thorough cleaning cycle in a different ultrasonic bath with fresh 99.9% isopropyl alcohol for 6 min. Following cleaning, specimens were dried with compressed air, and support structures were removed using a low-speed rotary instrument. Final polymerization was completed using a Xenon flashlight curing unit under protective gas for 7 min (2 × 2000 flashes, Otoflash G171-N2, NK Optik GmbH, Baierbrunn, Germany). Additionally, sixty cylinders were wet milled using a 5-axis milling machine (Ceramill Motion 2; Amann Girrbach, Koblach, Austria) from cross-linked polymethyl methacrylate (PMMA) provisional material (Telio CAD; Ivoclar Vivadent, Schaan, Liechtenstein) (Table 1) following the same dimensions described above. Milling tools with diameters of 1 and 2.5 mm (Roto RFID, Amann Girrbach, Koblach, Austria) were used to achieve the desired cylindrical shape. Cross-cut tungsten carbide burs (1958-012 Jet Tungsten Carbide Bur, Kerr, Brea, CA, USA) were used to separate the specimens from the disc and smooth out the attachment points. Following completion of the manufacturing process, both 3D printed and milled samples went through steam cleaning and visual inspection for any defects. Then, all specimens were polished under water cooling using a sequence of silicon carbide (SiC) papers of decreasing grit (500–800–1200–2000–4000, Struers, Ballerup, Denmark).

The 3D printed and milled samples were divided into three groups based on the surface treatment applied (Table 2). The control surface treatment was acid etching using 35% phosphoric acid (Transbond XT etching gel; 3M) for 30 s. The 3D printed samples were divided into the following groups: airborne particle abrasion (Group P-APA), rotary instrument roughening (Group P-RIR), and the control group treated with phosphoric acid (Group P-PAE). Similarly, the milled provisional materials were divided into airborne particle abrasion (Group M-APA), rotary instrument roughening (Group M-RIR), and the control group treated with phosphoric acid (Group M-PAE). Each group contained 20 samples. A guide, with a 6 mm in diameter circular window in its center, was designed and 3D printed in a similar fashion to the 3D printed provisional cylinders. The purpose of the guide is to sit on the cylinders, outlining the area to be surface treated in the center of the cylinder where the brackets will be bonded and aid the bonding process.

The surfaces of cylinders in Group 3D-APA and Group M-APA were treated using 50 μm aluminum oxide (Al_2_O_3_) airborne particle abrasion for 20 s, with a bar pressure of 2.5 and a distance of 10 mm. Subsequently, all surfaces were steam cleaned for 5 s at a distance of 50 mm, followed by a 5-minute cleaning in an alcohol ultrasonic bath and then air dried. All treatments were performed by a single investigator (L.A.A.).

The surfaces of cylinders in Group P-RIR and Group M-RIR were treated using an electric slow-speed rotary instrument (Ti-Max X; Henry Schein, Melville, NY, USA) at 5000 RPM. A wheel-shaped coarse grit (green) diamond bur (Maxima diamond bur 909-055C; Henry Schein) was used to apply 5 strokes at the center of the bonding surface. For standardization, all strokes were performed within 3 s and monitored using a timer. Since forces cannot be precisely controlled in the oral cavity, a timer was employed to ensure the required number of strokes were applied within the set time. A total of 10 burs were utilized, with each bur used for 4 samples before being replaced with a new one. All procedures were conducted by a single investigator (M.N.A.).

Following completion of mechanical surface treatments, the surfaces of all provisional material cylinders were etched with 35% phosphoric acid (Transbond XT etching gel; 3M, St. Paul, MN, USA) for 30 s, rinsed with a water spray for 20 s, and dried with compressed air for an additional 20 s. Then, primer (Transbond XT Primer; 3M, St. Paul, MN, USA) was applied to the surface of each provisional material cylinder and air-dried for 5 s to form an even layer. After which, stainless steel brackets for the maxillary central incisor (Victory Series; 3M, St. Paul, MN, USA) were bonded to provisional material cylinders using a light-cured adhesive (Transbond XT; 3M, St. Paul, MN, USA) applied to the bracket bases following the manufacturer’s instructions. The excess adhesive was removed using a dental explorer, and the adhesive was light-cured (Elipar; 3M, St. Paul, MN, USA) for 12 s on the mesial and distal sides of the bracket (6 s on each side), as recommended by the manufacturer. The bonding procedure for all brackets was performed by a single investigator (L.A.A.). After bonding the brackets, all samples were stored in distilled water at 37 °C for 24 h. To simulate the oral cavity environment, all samples underwent 5000 thermal cycles (Thermocycler model 1400, SD Mechatronik, Feldkirchen-Westerham, Germany) between 5 °C and 55 °C, with a dwell time of 30 s in an artificial saliva solution (Pickering Laboratories, Mountain View, CA, USA).

Three tests were used for the quantitative and qualitative evaluation of SBS and bond failure (Table 2). In quantitative evaluation, the sample size per group was calculated to be 17 with a total sample size of 102 using G-power software (G * Power 3.1.9.7, Heinrich-Heine-Universität Düsseldorf, Germany) [41] with an effect size of 1.305, power 0.95, and α 0.05. Therefore, the SBS test was conducted on 17 samples, which were randomly selected from each group following the guidelines of the International Organization for Standardization (ISO/TS 11405-2015) [42]. A custom apparatus was designed to mount the cylinders onto a universal testing machine (Instron 5965; Illinois Tool Works Inc., Glenview, IL, USA) and apply force through a knife-edge blade, flush and parallel to the interface of the bonding surfaces at a speed of 0.5 mm/min until bond failure occurred. The maximum force applied was recorded. The maximum force was displayed in the testing machine in Newton (N). The SBS was calculated into megapascal (MPa) by dividing the maximum force in N recorded by the surface area in square millimeters. For qualitative evaluation, two tests were performed pre- and post-SBS testing. In the pre-SBS testing, three samples from each group (*n* = 3 per group) were randomly selected after thermocycling for bonding interface evaluation using SEM (JEOL 6610LV series SEM; JEOL Ltd., Akishima City, Tokyo, Japan). These samples were mounted in acrylic resin and sectioned perpendicularly to the bracket-adhesive interface plane. The sectioned samples were wet ground using silicon carbide papers down to a 2500 mesh grit size and then polished with a 1 μm diamond slurry prior to SEM inspection. SEM analyses were performed in both secondary electron (SE) and backscattered electron (BSE) modes at magnifications of 30×, 100×, and 500×, with an accelerating voltage of 15 kV. To prepare the surfaces for SEM analysis, they were sputter-coated with an AuPd film. For post-SBS testing and evaluation of the bond failure interface, the de-bonded bracket bases and provisional material cylinders (*n* = 17 per group) were visually inspected, and images were captured using a digital microscope (Digital Microscope KH-7700; Hirox, Tokyo, Japan) at 50x magnification. The bond failure was classified according to the ARI score, which assesses the residual adhesive on the bonded area of the cylinder, where score 0 is given for samples showing no adhesive left on the cylinder, score 1 for samples showing less than 50% of adhesive left on the cylinder, score 2 for samples showing more than 50% of adhesive left on the cylinder and score 3 for samples with 100% of adhesive left on the cylinder with a distinct impression of the bracket mesh. All measurements from the images were conducted by a single blinded examiner (M.H.) using image editing software (2D Image File Viewing Software, KH-7700, Ver.2.10c; Hirox, Tokyo, Japan).

Two-way Analysis of Variance (ANOVA) was used to determine the effect of each variable (provisional crown material and surface treatment) on the SBS. The difference in the SBS between the 3D printed samples and milled provisional materials was statistically analyzed by employing one-way Analysis of Variance (ANOVA) followed by post hoc Tukey’s test. An independent sample *t*-test was used for intragroup comparison to determine the material/surface treatment combination that results in higher SBS. This analysis was conducted using the SPSS software (SPSS version 28.0; IBM, Armonk, NY, USA). The significance level chosen for this analysis was set at 0.05.

## 3. Results

### 3.1. Quantitative Evaluation Results

The mean, standard deviation, minimum and maximum SBS, and force at failure of 3D printed and milled provisional materials, subjected to three different surface treatments: rotary instrument roughening, airborne particle abrasion, and phosphoric acid etching measured and expressed in MPa and N (Table 3). Mean de-bond load values decreased in the following order: P-RIR > P-APA > P-PAE > M-APA > M-RIR > M-PAE. Two-way ANOVA was used to determine the effect of each variable (provisional crown material and surface treatment) on the SBS. A statistically significant difference was found for both provisional crown material and surface treatment (*p* < 0.001) on the SBS of orthodontic brackets to provisional crown material.

A one-way ANOVA test was performed to determine if there were statistically significant differences in the mean SBS values among the groups, and a significant difference was found. Further pairwise comparisons of the different surface treatments applied to the two types of provisional restorations were conducted using the Tukey HSD test. The results showed statistically significant differences in the mean SBS for most comparisons, except for the following combinations: Group P-PAE and Group M-APA (*p* = 0.309), Group M-RIR and Group M-APA (*p* = 0.220), lastly Group M-RIR and Group M-PAE (*p* = 0.958) (Table 3).

Within the group of provisional crown materials that were 3D printed, there were statistically significant differences among all surface treatments (Table 4). The rotary instrument treatment achieved the highest SBS values (mean = 12.709 MPa), followed by airborne particle abrasion (mean = 9.367 MPa) and the control group (mean = 7.778 MPa). This finding indicates that rotary instrument roughening can produce a higher SBS orthodontic brackets to 3D printed provisional crown material. Within the milled provisional crown materials group, there was no statistically significant difference between the rotary instrument surface treatment and the control group (*p* = 0.301). However, there were statistically significant differences between airborne particle abrasion and rotary instrument surface treatment (*p* = 0.001) and between airborne particle abrasion and the control group (*p* < 0.001) (Table 4). Airborne particle abrasion achieved the highest SBS values with milled materials (mean = 6.932 MPa) compared to rotary instrument surface treatment (mean = 6.01 MPa) and the control group (mean = 5.665 MPa). This finding indicates that airborne particle abrasion can produce a higher SBS of orthodontic brackets to milled provisional crown material.

### 3.2. Qualitative Evaluation Results

The nature of the bond failure was examined under a digital microscope using the ARI index. In 3D printed materials, more samples received a score of 1, indicating that less than fifty percent of the adhesive remained on the surface of the provisional material (Table 5 and Table S1). For samples treated with airborne particles, none received a score of 0, suggesting that residual adhesive remained on all samples. In contrast, many samples from the milled materials group received a score of 0, indicating that no adhesive was left on the sample surfaces. All samples in the milled control group had an adhesive fracture (ARI score 0) between the provisional material and adhesive. Cohesive failure between the bracket and adhesive was less common in milled provisional materials compared to 3D printed samples. Lower ARI scores may lead to de-bonding during treatment, requiring re-bonding and potentially increasing treatment time. Higher ARI scores may cause enamel cracks or surface damage during de-bonding, and the remaining adhesive must be removed using rotary instruments.

**Figure 1:**
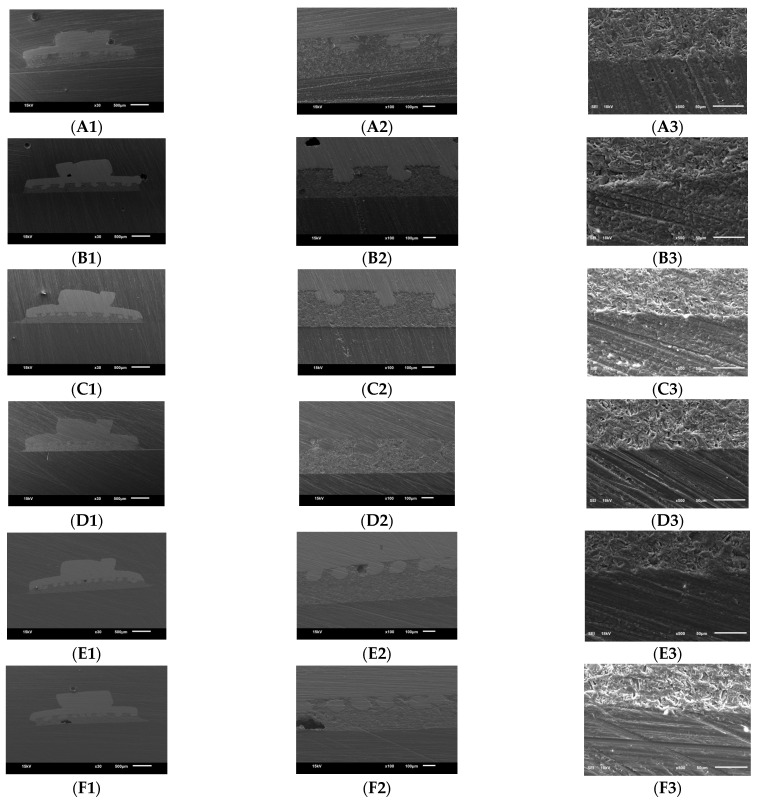
SEM images of one representative sample from each group showing the bonding interface in three magnifications: 30×, 100×, and 500×. (**A1**) group P-APA ×30, (**A2**) group P-APA ×100, (**A3**) group P-APA ×500, (**B1**) group P-RIR ×30, (**B2**) group P-RIR ×100, (**B3**) group P-RIR ×500, (**C1**) group P-PAE ×30, (**C2**) group P-PAE ×100, (**C3**) group P-PAE ×500, (**D1**) group M-APA ×30, (**D2**) group M-APA ×100, (**D3**) group M-APA ×500, (**E1**) group M-RIR ×30, (**E2**) group M-RIR ×100, (**E3**) group M-RIR ×500, (**F1**) group M-PAE ×30, (**F2**) group M-PAE ×100, (**F3**) group M-PAE ×500. Provisional material samples treated with RIR (**B**) (**E**) exhibited more pronounced and deeper surface irregularities than other groups, with milled samples showing visible cracks and wider surface details than 3D printed samples. Samples treated with APA (**A**) (**D**) displayed numerous homogeneous and shallow surface irregularities. The control groups (**C**) (**F**) had a relatively smoother surface. The surface roughness in SEM analysis decreases in the following order: 1E.M-RIR > 1B. P-RIR > 1A. P-APA > 1D. M- APA > 1C. P-PAE > 1F.M-PAE.

The SEM analysis of the bracket-adhesive interface was conducted at three magnifications (30×, 100×, and 500×). The airborne treated materials displayed a rough surface with numerous small features and evenly distributed pores, especially in the 3D printed samples compared to the milled APA-treated ones (Figure 1A,D). The rotary-treated samples had a more irregular surface with larger and more unevenly distributed features (Figure 1B,E). M-RIR samples showed large, unevenly distributed pores and visible cracks, with high contrast regions indicating variations in composition (Figure 1E). The control group had a smoother surface compared to the other groups, with fewer visible pores and a more uniform texture (Figure 1C,F). Milled provisional crown samples showed microscale irregularities compared to their corresponding 3D-printed samples, with M-RIR samples showing more surface irregularities.

## 4. Discussion

The SBS of orthodontic brackets to 3D printed and milled provisional materials was measured following three surface treatments: airborne particle abrasion (APA), rotary instrument roughening (RIR), and phosphoric acid etching (PAE). SBS was measured in megapascals (MPa), revealing significant differences among provisional materials and treatments. Therefore, the null hypothesis of the present study was rejected.

Orthodontic treatment frequently lasts over a year, during which the bond strength of brackets can diminish over time [23,32]. This bond strength is crucial for the success of the treatment. Different factors can affect the bonding of orthodontic brackets to teeth or crowns. For example, intraoral degradation of adhesive resin, fluctuation in temperature and pH, masticatory forces, and microbial degradation [22]. According to the International Organization for Standardization (ISO), oral conditions can be simulated “in vitro” using an aging procedure with a thermocycling machine. An appropriate aging simulation involves performing 500 cycles in water, with temperatures alternating between 5 °C and 55 °C and a 5-second interval between each cycle (ISO 11405) [42,43]. However, this number of cycles is likely insufficient to achieve a “real” simulation [22,44]. Therefore, to simulate the aging process, the samples in this study underwent thermocycling for 5000 cycles, between 5 °C and 55 °C, with a 30-second dwell time and were then stored in distilled water at 37 °C for 24 h. The use of 5000 cycles is a standard benchmark for testing the durability of provisional restorations and is considered adequate for approximating the stress experienced over their typical clinical lifespan. This can be justified by the fact that the teeth are subject to thermal or mechanical forces during various activities like eating, drinking, etc., which may account for around 20–50 thermal cycles per day, and the duration of the treatment also varies from weeks to months [43].

The optimal bond strength, essential for effective tooth movement, between orthodontic brackets and provisional crown materials is typically between 6 and 8 MPa [22,37,38]. In this study, significant differences in mean SBS values among the groups were observed, consistent with findings from other studies [34]. The 3D-printed provisional crowns exhibited significantly higher SBS than their milled counterparts. Both materials showed that the lowest bond strength is primarily associated with phosphoric acid treatment. The results of 3D printed materials with rotary instrument treatment yielded the highest SBS values (12.7 ± 2.37 MPa), followed by airborne particle abrasion (9.3 ± 0.90 MPa), and phosphoric acid etching (7.7 ± 0.83 MPa). In the milled provisional crown material groups, airborne particle abrasion achieved the highest SBS values (6.9 ± 0.60 MPa), followed by rotary instrument treatment (6.0 ± 0.74 MPa) and phosphoric acid etching (5.6 ± 0.65 MPa). Significant differences in mean SBS values were observed among groups, with exceptions between P-PAE and M-APA (*p* = 0.309) and between M-RIR and both M-APA (*p* = 0.220) and M-PAE (*p* = 0.958).

The subtractive milling technique has several reported disadvantages: it leads to raw material wastage, the milling tools have a limited lifespan due to wear and abrasion, and the precision of the milled material is influenced by the movement, type and size of the tool, resulting in a poorer fit and marginal adaptation for complex designs [5,10]. Conversely, 3D printing offers significant advantages, such as the ability to create restorations with intricate internal geometries and complex prostheses while minimizing material waste [18,19]. However, milled materials typically have a higher filler content because they are processed under higher temperatures and pressure, leading to lower porosity, fewer voids, and reduced residual monomers compared to 3D printing and conventional resins [9].

Although the enhanced bond strength of surface-treated printed provisional crowns may facilitate orthodontic tooth movement, clinicians should exercise caution during the de-bonding process, as bracket removal may compromise the integrity of the underlying printed crown. The present study also demonstrated greater resin cement retention on the 3D printed substrate, which was not seen with milled samples. Nevertheless, more damage is expected on the underlying 3D-printed provisional crowns. While the crowns in this study are intended for long-term provisional use, the need for replacement in the case of definitive prostheses should be anticipated following the de-bonding of the orthodontic brackets [38,45]. High MPa values provide long-term durability against orthodontic forces but may pose difficulty in the removal of brackets, causing enamel damage.

The results of the present study are in alignment with the findings of Soliman et al. [31] despite the use of different bracket materials and adhesive systems in both studies. The results indicate superior SBS of orthodontic brackets to airborne particle-abraded milled CAD/CAM provisional crowns. Similarly, Haber et al. [32] investigated the effects of various surface treatments on the SBS of metal brackets to milled CAD/CAM provisional crowns. They found that sandblasting alone produced the highest bond strength. However, when mechanical roughening was combined with a plastic conditioner, bond strength improved, with no significant difference between sandblasted and diamond bur roughened surfaces. One study examined the effect of surface treatment on the SBS of metal brackets to 3D-printed provisional crowns. In that study, sandblasting with 50 µm aluminum oxide and 30 µm silica-coated alumina particles was used. Both treatments resulted in higher bond strength in milled CAD/CAM crowns compared to the control group, although the difference was not statistically significant [34]. In contrast, our findings differ from those of Alhendi and Alassiri [35], who studied the effects of surface treatments on milled crowns. However, their samples were not subjected to thermocycling, making a direct comparison to our study inapplicable.

The residual adhesive after de-bonding was evaluated using the ARI through digital microscope analysis. The results indicated that there were more cohesive failures in 3D printed materials compared to milled materials [32]. In the 3D printed groups, many samples retained more adhesive on the surface, while milled samples often had no adhesive remaining, suggesting adhesive fractures. This difference could be due to microporosities produced by rotary instruments or airborne particle abrasion, which enhance the bonding of brackets to 3D-printed provisional crowns. All samples in the milled control group showed adhesive failures (ARI score of 0), indicating weak bond strength, consistent with findings from Borzangy [30] and Shahin et al. [39].

SEM analysis of the bracket-adhesive interface revealed distinct surface morphologies among the groups. Samples treated with a rotary instrument displayed irregular, uneven surfaces, while milled samples showed larger pores and visible cracks. Airborne particle-abraded materials had rough surfaces with numerous small features, particularly in the 3D-printed samples. The control group had smoother surfaces with fewer visible pores and a more uniform texture. In comparison, milled provisional crown samples showed microscale irregularities when compared to 3D printed counterparts, with M-RIR (Milled-Rotary instrument roughened) samples displaying even more surface irregularities. The mechanical interlocking has been increased, resulting from the formation of deep craters and streaks, significantly contributing to bond strength in rotary roughened surfaces [22,39,40].

A major limitation of the present study is its in vitro design. Although efforts were made to simulate the oral environment through thermocycling and standardized mechanical SBS testing, certain factors, such as the presence of saliva and the fluctuating pH levels, could not be replicated. Additionally, the CAD/CAM materials were fabricated as cylindrical specimens with thicknesses greater than those typically used in clinical dental prostheses, potentially affecting the generalizability of the findings. Furthermore, the de-bonding forces in this study were primarily applied at the interface between the base of the bracket and the underlying CAD/CAM milled or printed substrates, which does not accurately reflect the complex stresses generated during orthodontic treatment with arch wires. The study could have evaluated the comparison of other surface treatments, like chemical surface treatments.

## 5. Conclusions

Based on the study, the following conclusions were made:

1. Different mechanical surface treatments enhance the bonding of orthodontic brackets to the surface of provisional crowns. The highest SBS for 3D printed provisional crowns was achieved with diamond bur rotary instrument roughening, while the highest SBS for milled provisional crowns was achieved with Al2O3 airborne particle abrasion;
2. The 3D-printed provisional crowns had higher bond strength compared to milled provisional crowns.

## 6. Clinical Significance

The results indicated that 3D-printed provisional crowns exhibited higher bond strength compared to milled provisional crowns. Furthermore, the mechanical surface treatments enhanced the SBS of metallic orthodontic brackets to CAD/CAM provisional crowns. These findings can guide clinicians in selecting the most effective surface treatment to enhance the bonding strength of provisional restorations, ultimately improving their performance and longevity in dental applications.

## Figures and Tables

**Table 1:** The manufacturer of the 3D-printed and milled PC material used in the study.

Material	Manufacturer	Technology	Ref No./Lot No.	Indication
Detax Freeprint Temp	DETAX GmbH, Ettlingen, Germany	DLP 3D printing	04063/251210	Temporary crowns and bridges and implant-supported restorations
Telio CAD	Ivoclar Vivadent, Schaan, Liechtenstein	Milling	686310/YBB1TX	Temporary crowns and bridges and implant-supported restorations

**Table 2:** Table showing the number of samples and distribution. (APA): Air particle abrasion (RIR): Rotary instrument roughening (PAE): Phosphoric acid etching (SBS): Shear bond strength (SEM): Scanning electron microscopy.

Provisional Crown Material	Surface Treatment	Quantitative Evaluation	Qualitative Evaluation
3D Printed (P) (*n* = 60)	APA (*n* = 20)	SBS Testing (*n* = 17)	Microscopic Inspection (*n* = 17)
			SEM Evaluation (*n* = 3)
	RIR (*n* = 20)	SBS Testing (*n* = 17)	Microscopic Inspection (*n* = 17)
			SEM Evaluation (*n* = 3)
	PAE (*n* = 20)	SBS Testing (*n* = 17)	Microscopic Inspection (*n* = 17)
			SEM Evaluation (*n* = 3)
Milled (M) (*n* = 60)	APA (*n* = 20)	SBS Testing (*n* = 17)	Microscopic Inspection (*n* = 17)
			SEM Evaluation (*n* = 3)
	RIR (*n* = 20)	SBS Testing (*n* = 17)	Microscopic Inspection (*n* = 17)
			SEM Evaluation (*n* = 3)
	PAE (*n* = 20)	SBS Testing (*n* = 17)	Microscopic Inspection (*n* = 17)
			SEM Evaluation (*n* = 3)

**Table 3:** Showing load, in Newtons (N), and SBS, in Megapascals (MPa), data for all groups (mean, standard deviation, min–max), and statistical significance based on the Tukey HSD test.

Material	Surface Treatment	Group * (*n* = 17)	Mean ± (SD) Load (N) SBS (MPa)	Minimum Load (N) SBS (MPa)	Maximum Load (N) SBS (MPa)
3D Printed	Airborne Particle Abrasion	P-APA	112.311030 ± (10.8248359)	93.0441	135.5667
			9.367058 ± (0.9028220)	7.7601	11.3067
	Rotary Instrument	P-RIR	152.375986 ± (28.4292384)	121.1172	208.1122
			12.708589 ± (2.3710791)	10.1015	17.3572
	Phosphoric Acid Etching	P-PAE ^a^	93.255451 ± (10.0156765)	69.9952	107.5571
			7.777769 ± (0.8353358)	5.8378	8.9706
Milled	Airborne Particle Abrasion	M-APA ^a,b^	83.109824 ± (7.2085101)	66.5114	94.6085
			6.931595 ± (0.6012102)	5.5472	7.8906
	Rotary Instrument	M-RIR ^b,c^	72.050228 ± (8.8743982)	57.5604	86.5924
			6.009193 ± (0.7401500)	4.8007	7.2220
	Phosphoric Acid Etching	M-PAE ^c^	67.926420 ± (7.9071826)	51.8089	83.3611
			5.665256 ± (0.6594814)	4.3210	6.9526

* Groups with the same superscript letter show no statistically significant difference.

**Table 4:** Tukey’s HSD test for intragroup provisional crown material surface treatment comparison.

Surface Treatment Comparison		3D Printed Provisional Crown Material	Milled Provisional Crown Material
		*p*	*p*
Rotary Instrument	Airborne Particle Abrasion	<0.001	0.001
Airborne Particle Abrasion	Phosphoric Acid Etching	0.012	<0.001
Phosphoric Acid Etching	Rotary Instrument	<0.001	0.301

**Table 5:** ARI scores the samples with their frequency and percentage according to digital microscope analysis.

ARI Score *	Number of Samples (Percentage) in Each Group					
	P-APA	P-RIR	P-PAE	M-APA	M-RIR	M-PAE
**0**	0 (0%)	7 (41.2%)	1 (5.9%)	10 (58.8%)	16 (94.1%)	17 (100%)
**1**	8 (47.1%)	6 (35.3%)	9 (52.9%)	6 (35.3%)	0 (0%)	0 (0%)
**2**	5 (29.4%)	1 (5.9%)	4 (23.5%)	1 (5.9%)	1 (5.9%)	0 (0%)
**3**	4 (23.5%)	3 (17.6%)	3 (17.6%)	0 (0%)	0 (0%)	0 (0%)

* ARI score 0 = no adhesive left on the cylinder, 1 = less than half of the adhesive left on the cylinder, 2 = more than half of the adhesive left on the cylinder, 3 = all adhesives left on the cylinder with a distinct impression of the bracket mesh.

## Data Availability

Data could be available upon reasonable request to the corresponding author.

## Supplementary Materials

The following supporting information can be downloaded at: https://www.mdpi.com/article/10.3390/jfb15120358/s1, Figure S1: study design; Table S1: ARI scores for each sample.
