# Supplementary material for: Effect of Mechanical Surface Treatment on Shear Bond Strength of Orthodontic Brackets to 3D Printed and Milled CAD/CAM Provisional Materials: An In Vitro Study
Source: J Funct Biomater. 2024 Nov 25;15(12):358. doi: 10.3390/jfb15120358 (PMC11728244; doi:10.3390/jfb15120358)
Supplement: Supplementary file 1 [file jfb-15-00358-s001.zip › Table S1. ARI scores for each sample .pdf]

Supplementary Table S1. ARI scores for each sample.

Sample #	**1**	**2**	**3**	**4**	**5**	**6**	**7**	**8**	**9**	**10**	**11**	**12**	**13**	**14**	**15**	**16**	**17**
**3D printed samples**																	
P-APA	1	3	2	2	1	2	3	1	1	2	3	3	1	2	1	1	1
P-RIR	0	0	0	1	2	0	3	0	0	3	3	1	1	0	1	1	1
P-PAE	1	2	1	1	1	1	1	3	3	0	2	1	1	1	3	2	2
**Milled samples**																	
M-APA	0	2	1	0	0	1	0	0	0	0	0	0	0	1	1	1	1
M-RIR	0	0	0	0	0	2	0	0	0	0	0	0	0	0	0	0	0
M-PAE	0	0	0	0	0	0	0	0	0	0	0	0	0	0	0	0	0
