# Supplementary figures and images for: Effect of Mechanical Surface Treatment on Shear Bond Strength of Orthodontic Brackets to 3D Printed and Milled CAD/CAM Provisional Materials: An In Vitro Study
Source: J Funct Biomater. 2024 Nov 25;15(12):358. doi: 10.3390/jfb15120358 (PMC11728244; doi:10.3390/jfb15120358)

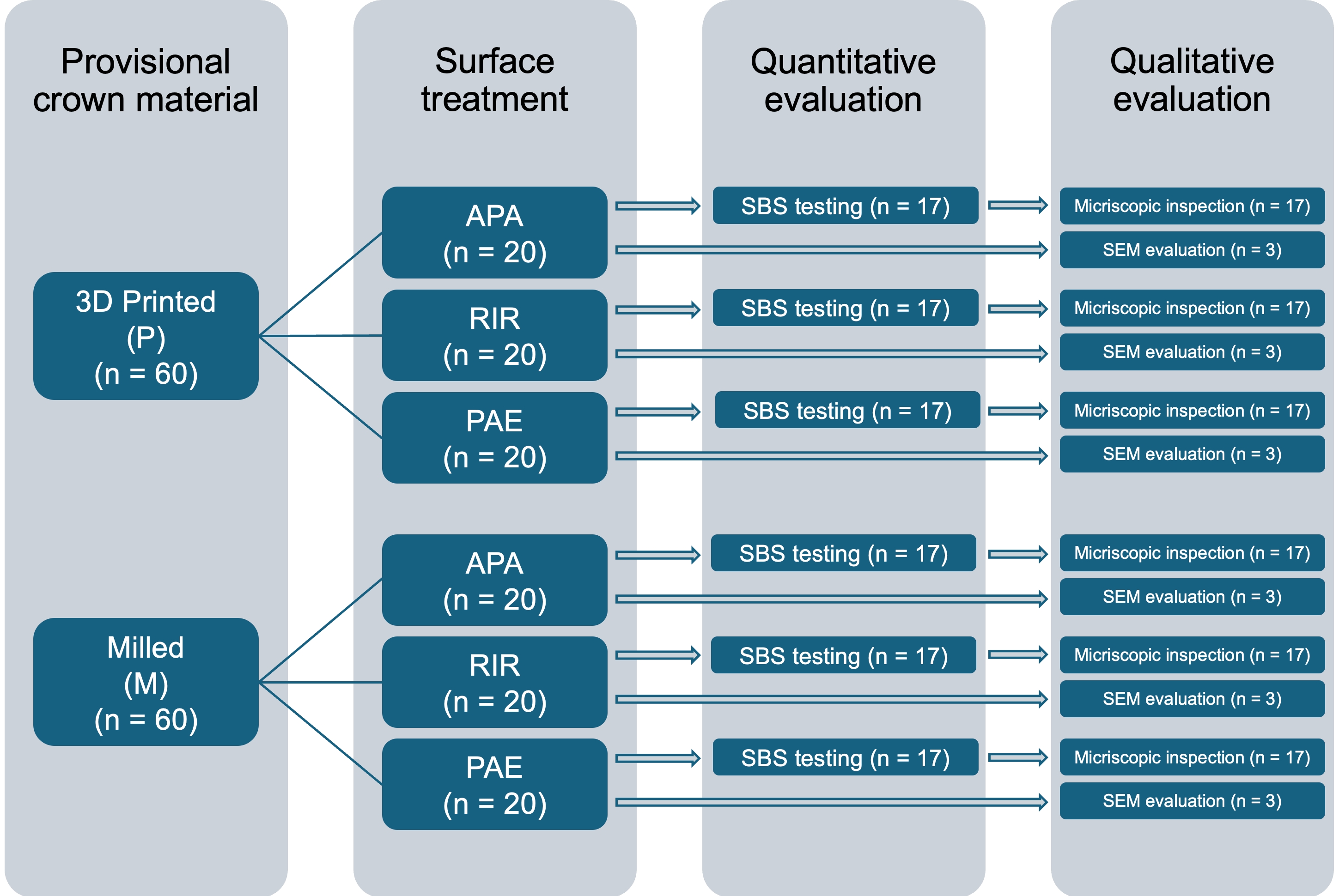

Supplement: Supplementary file 1 [file jfb-15-00358-s001.zip › Figure S1. Study design.jpg]
